# How Does Inter-Epitope Spacer Variation Within Artificial Immunogens Based on T-Cell Epitopes of Tick-Borne Encephalitis Virus Affect Immunogenicity?

**DOI:** 10.3390/vaccines14090770

**Published:** 2026-09-02

**Authors:** Elena V. Yakovleva, Denis V. Antonets, Mariya B. Borgoyakova, Ekaterina V. Starostina, Vladimir A. Yakovlev, Elizaveta V. Shaburova, Denis N. Kisakov, Lyubov A. Kisakova, Olga Y. Volkova, Nadezhda B. Rudometova, Andrey P. Rudometov, Larisa I. Karpenko

**Affiliations:** 1 State Scientific Center of Virology and Biotechnology “Vector”, Rospotrebnadzor, 630559 Koltsovo, Russia; 2 Institute for Artificial Intelligence, Lomonosov Moscow State University, 119234 Moscow, Russia; 3 Institute of Molecular and Cellular Biology SB RAS, 630090 Novosibirsk, Russia

**Keywords:** multi-epitope, spacer, tick-borne encephalitis virus, T-cell immunogen, immunogenicity, T-cell response, DNA vaccine

## Abstract

**Background/Objectives:** In recent years, researchers have directed considerable attention toward the activation of the T-cell response in the development of vaccines against viral infections, including the tick-borne encephalitis virus (TBEV). A particularly promising approach to developing effective and safe T-cell vaccines involves the use of artificial multi-epitope immunogens. The selection of spacers that link epitopes within a construct can have a significant effect on the immunogenicity of multi-epitope constructs. The objective of this study was to design multi-epitope TBEV immunogens using various spacers and to evaluate their immunogenicity. **Methods:** The present study involved the design of three multi-epitope immunogens, which were developed based on T-cell epitopes from TBEV proteins. The AG1-ub construct contained optimized alanine spacers, the AG2-ub construct contained GPGPG spacers, and the AG4-ub construct contained no spacers. Three DNA vaccines encoding the designed immunogens were subsequently produced. To assess the immunogenicity of the constructs, BALB/c mice were immunized with the designed DNA vaccines via electroporation. **Results:** The ELISpot assay demonstrated that DNA vaccines encoding AG1-ub and AG4-ub induced a significant number of IFN-γ-producing cells. The DNA vaccine encoding AG2-ub, a multi-epitope with GPGPG spacers, exhibited low immunogenicity, potentially attributable to inadequate processing or misfolding of the AG2-ub protein, thereby affecting the molecule’s structure. **Conclusions:** The data provided in this study on the effect of spacers on the immunogenicity of multi-epitope constructs can be used to optimize the design of vaccine candidates. However, further research is needed to assess their clinical potential.

## 1. Introduction

The development of next-generation antiviral vaccines remains a significant area of research. The development of immunogens capable of providing long-lasting protective immunity is a rather complex task. Recently, particular attention in this area has been focused on the development of multi-epitope T-cell immunogens.

Artificial multi-epitope antigens represent a promising approach to the development of effective and safe vaccines that elicit T-cell immune responses. Their key advantage over conventional vaccine types is their ability to combine epitopes from a wide array of viral proteins into a single molecule. In recent years, a plethora of studies on the design of multi-epitope immunogens have been published [1,2,3,4,5]. A number of multi-epitope immunogens, such as HIV-1 immunogens, have advanced to clinical trials [6,7,8].

However, the development of T-cell multi-epitopes is associated with a number of challenges. The choice of an algorithm for the design and assembly of the selected epitopes, which is intended to ensure maximum efficacy, necessitates the consideration of several factors. It is known that the immunogenicity of multi-epitope antigens is determined by many factors, such as epitope selection, codon optimization, and the choice of spacers connecting the epitopes within the construct [9,10].

Spacers are specialized sequences that connect the domains of synthetic proteins to one another. Numerous studies have examined the effect of spacers on the function of chimeric/hybrid proteins [11,12,13]. It is imperative to acknowledge that the objective of these studies is to address issues such as the prevention of the emergence of undesirable immunogenic epitopes, where two target peptides combine within the recombinant polyprotein. To minimize these occurrences and improve the affinity of the selected fragments for the transporter associated with antigen processing (TAP), as well as promote the desired proteasomal and immunoproteasomal processing of the antigen, epitopes are typically flanked by spacers, which are short amino acid motifs. There are two types of spacers: rigid and flexible [14]. Thus, rigid spacers can either form a stable two-dimensional (2D) structure, such as (EAAAK)n, which forms an alpha-helix, or disrupt the structure by isolating two domains from each other, such as proline-rich spacers combined with alanine, lysine, or glutamic acid. Flexible spacers are characterized by the greater rotational freedom of their peptide bonds, and they often contain a high proportion of glycine, as is the case with the GGGS spacer. These two types of sequences are found in known multi-epitope vaccines [15,16], though more specific ones exist as well, such as the AAY spacer [17].

The effect of inter-epitope spacers on the immunogenicity of a construct is a topic that has remained understudied. Nevertheless, a considerable number of researchers are confined to theoretical predictions of model proteins or neglect to compare alternative strategies within a single model system [15,16,17].

In this regard, the acquisition of diverse experimental data concerning T-cell epitopes, various strategies for designing multi-epitope constructs, and the efficacy of artificial multi-epitope immunogens derived from them will substantially enhance our comprehension of this field and improve the predictive capabilities of rational design programs. The data obtained can also be used in the future to develop effective antiviral vaccines.

For the present study, the tick-borne encephalitis virus (TBEV) multi-epitope immunogen was selected as the model object for a number of reasons, which are outlined below.

TBEV is classified within the *Flaviviridae* family. Until recently, it was officially designated as *Orthoflavivirus encephalitidis*. According to the current ICTV classification (MSL #41), it has been divided into two species and named *Orthoflavivirus zilberi* (which combines the Far Eastern, Siberian, and Baikal subtypes of TBEV) and *Orthoflavivirus neudoerflense* (which includes the European subtype of TBEV). TBEV poses a significant public health concern. Annually, approximately 10,000–15,000 individuals contract the virus [18]. The increasing incidence of TBEV infections, the expansion of the virus’s range, and its mutability necessitate the enhancement of TBEV prevention strategies through the development of novel vaccines and the refinement of existing ones [19,20].

The currently licensed vaccines, which are based on inactivated TBEV, elicit a protective immune response; however, these vaccines require multiple doses. These vaccines primarily elicit a humoral immune response, but neutralizing antibodies are only detected in the serum of vaccinated individuals for a period of 5–10 years. One potential explanation for the limited duration of this immunity is the weak T-cell response elicited by these vaccines [21,22,23,24].

To date, there are only a few studies in the literature concerning the development of multi-epitope vaccines against TBEV, and even these are limited to theoretical modeling [25,26]. However, to date, there have been no studies that have systematically compared different spacer selection strategies within a single set of TBEV epitopes using a DNA vaccine platform in a BALB/c mouse model.

The present study provides the results of the design and immunogenicity assessment of DNA vaccines encoding TBEV multi-epitope immunogens, constructed using alanine-containing or GPGPG spacers, or without spacers.

## 2. Materials and Methods

### 2.1. Bacterial Strains, Viruses, Cell Cultures, and Plasmids

The *E. coli* strain Stbl3 (Invitrogen, Waltham, MA, USA) was used to produce plasmid DNA.

The pVAX1 vector was used for cloning (Invitrogen, Waltham, MA, USA).

A HEK293 cell culture was used to analyze the expression of target genes (cell culture collection of the FBIS SSC VB «Vector» Rospotrebnadzor, Koltsovo, Russia).

### 2.2. Epitope Identification

The selection of T-cell epitopes was conducted using data derived from experiments involving the immunization of mice with flaviviruses, which are available in the Immune Epitope Database (IEDB) 2.22 [27]. Subsequently, the epitopes obtained were aligned against the TBEV proteome (UniProtKB—Q01299). Further selection was limited to peptides with an identity level of more than 70% (with a maximum of five amino acid substitutions, insertions, or deletions). Oligopeptides, which consist of clusters of overlapping epitopes in native viral proteins, were prioritized for inclusion in the vaccine. The inclusion of these clusters has been shown to enhance the overall immunogenicity of the constructs [28]. Thereafter, an analysis was conducted of CTL and HTL epitopes restricted by various alleles of mouse major histocompatibility complex (MHC) class I and II molecules, using the following tools: NetMHCpan-4.1 and NetMHCIIpan-4.0 [29] (access date—14 March 2023). The analysis was performed on the alleles of the BALB/c mouse strain (H-2^d^ haplotype): H-2-Kd, H-2-Dd, H-2-Ld (class I) and H-2-IAd, H-2-IEd (class II). Predictions were made for standard-length peptides: the 9-mer motif is identified for MHC class I, while the 15-mer is identified for MHC class II, including the core 9-mer motif. The peptides were ranked by percentile rank (%Rank). Peptides with a %Rank ≤ 0.5% were classified as strong binders, while those with a %Rank ≤ 2.0% were designated as weak binders. All other prediction parameters were set to their default values. The final design of multi-epitope immunogens included the selection of peptide fragments that demonstrated the greatest capability to bind to diverse MHC alleles.

### 2.3. Design of Immunogens

Multi-epitope immunogens were designed using the PolyCTLDesigner software (version 0.3) [30]. In order to investigate the effect of different types of inter-epitope spacers on the immunogenicity of multi-epitope constructs, three immunogens were designed. For the first immunogen (AG1-ub), the PolyCTLDesigner software was used to select alanine-containing spacer sequences between epitopes. For the second immunogen (AG2-ub), the sequence GPGPG was selected as the spacer. The third immunogen (AG4-ub) consisted of a sequence in which the epitopes were linked directly without the use of spacers. The order and arrangement of the epitopes within the constructs were identical for all constructs. Ubiquitin was added to the N-terminus of all constructs, and the C-terminus was tagged with the marker epitope EPFRDYVDRFYKTLR, recognized by the monoclonal antibody 29F2, and the universal Th epitope PADRE—AKFVAAWTLKAAA.

The GeneDesigner 3.0 software [31] was then used to design artificial gene sequences based on the developed multi-epitope constructs. Codon optimization was performed using the JCat web service [32]. The *Mus musculus* species was selected as the host organism. The optimization of codons was executed in accordance with the codon usage patterns that are characteristic of genes that are highly expressed in mouse cells. The objective of this process was to maximize the efficiency of target protein translation in mouse cells. The GC content of the resulting sequences was approximately 56%. In order to perform directed cloning into the pVAX1 plasmid vector, a PspLI restriction site was inserted at the 5′-end of the synthetic genes, and a PspOMI site was inserted at the 3′-end.

### 2.4. Structural Modeling and Theoretical Evaluation of the Immunogen Physicochemical Properties

ExPASy ProtParam (https://web.expasy.org/protparam/, accessed on 15 November 2025) was used to calculate the chemical and physical properties of the protein sequence, including the theoretical isoelectric point (pI), the instability index, and the grand average of hydropathy (GRAVY). The Protein Structure Prediction Server (PSIPRED) v4.0 (https://bioinf.cs.ucl.ac.uk/psipred, accessed on 17 November 2025) and the AlphaFold Server (https://alphafoldserver.com/welcome, accessed on 20 November 2025) were used to predict the secondary and tertiary structures of target proteins, respectively.

### 2.5. Development of the Plasmids pVAX-AG1-ub, pVAX-AG2-ub, and pVAX-AG4-ub

The nucleotide sequences (ag1-ub, ag2-ub, and ag4-ub) were synthesized and cloned into the pVAX1 plasmid vector (Invitrogen, Waltham, MA, USA). The cloning process was carried out using restriction endonucleases PspLI and PspOMI (SibEnzyme, Novosibirsk, Russia). The resulting plasmids were designated pVAX-AG1-ub, pVAX-AG2-ub, and pVAX-AG4-ub, respectively. The structure of the recombinant plasmids obtained was confirmed by Sanger sequencing at the “Genomics” Shared-Use Center of the SB RAS (Novosibirsk, Russia). The analysis was performed using the CEQ2000 Dye Terminator Cycle Sequencing Kit (Applied Biosystems, Waltham, MA, USA) and the ABI 3130xl 16-capillary automated sequencer (Applied Biosystems, Waltham, MA, USA). The SnapGene 3.2.1 software (GSL Biotech LLC, Chicago, IL, USA) was used to align the nucleotide sequences.

### 2.6. Production of Recombinant Plasmids

Competent *E. coli* Stbl3 cells (Invitrogen, Waltham, MA, USA) were transformed with the plasmids pVAX-AG1-ub, pVAX-AG2-ub, and pVAX-AG4-ub. To competent *E. coli* Stbl3 cells, 50 ng of plasmid DNA was added, and the mixture was then incubated on ice for 40 min. Subsequently, the cells were exposed to a “heat shock” treatment at a temperature of 42 °C for a duration of 40 s. The cells were cooled on ice for 5 min, then 275 μL of LB medium was added to the mixture, which was then incubated at a temperature of 37 °C for a duration of 60 min. At the end of the incubation, the transformed cells were plated onto a Petri dish containing LB agar medium (LB medium with 1.5% agar) supplemented with an antibiotic (kanamycin 100 μg/mL).

Subsequently, 10 mL of an overnight culture of *E. coli* Stbl3 cells that had been transformed with the plasmids was added to 40 mL of LB medium containing 100 μg/mL kanamycin. The culture was incubated at 37 °C until the optical density at 600 nm (OD600) reached 1.0. Subsequently, 2.5 mL of the culture was added to each of 18 flasks containing 150 mL of LB medium with 100 μg/mL kanamycin. The cultures were then incubated at 37 °C overnight. Subsequently, the cells were harvested by centrifugation for 20 min at 7000 rpm on an Avanti JXN-30 centrifuge at 4 °C (Beckman Coulter, Brea, CA, USA).

To isolate plasmid DNA, the QIAGEN EndoFree Plasmid Purification Giga Kit (Qiagen, Hilden, Germany) was used. DNA isolation was performed according to the manufacturer’s protocol.

### 2.7. Transfection of HEK293 Cells

To analyze the expression levels of the ag1-ub, ag2-ub, and ag4-ub genes, HEK293 cell cultures (cell culture collection of the FBIS SSC VB «Vector» Rospotrebnadzor, Koltsovo, Russia) were transfected with the pVAX-AG1-ub, pVAX-AG2-ub, and pVAX-AG4-ub plasmids. The HEK293 cells were cultivated at a temperature of 37 °C, with relative humidity set at 80% and a CO_2_ concentration of 5%. The growth medium was composed of DMEM, 10% heat-inactivated (30 min at 56 °C) fetal bovine serum (FBS), 2 mM L-glutamine, and the antibiotic gentamicin (50 μg/mL). To achieve 85–100% confluence of the cell monolayer at the moment of use (24 h), the seeding concentration was 1 × 10^5^ cells/cm^2^. The degree of cell monolayer confluence was monitored using an inverted microscope at low magnification. The transfection process was performed using Lipofectamine 3000 (Thermo Fisher Scientific, Waltham, MA, USA), according to the manufacturer’s protocol.

### 2.8. Determination of mRNA Expression Levels Using Reverse Transcription Polymerase Chain Reaction (RT-PCR)

The transcription efficiency of DNA plasmids in HEK293 cells was assessed through a series of analytical procedures. Initially, total mRNA was isolated, and subsequently, complementary DNA (cDNA) was obtained by RT-PCR. The reaction was performed using the specific primers pVAX-Uni-F (5′-TCGAAATTAATACGACTCACTATAGGGAG-3′) and pVAX-Uni-R (5′-CTGGCAACTAGAAGGCACAG-3′).

The RNA was isolated from HEK293 cell cultures using the “LIRA+ for RNA, DNA, and Protein Isolation” kit (Biolabmix, Novosibirsk, Russia), according to the manufacturer’s protocol. RT-PCR was performed using the “BioMaster RT-PCR–Extra (2×)” kit (Biolabmix, Novosibirsk, Russia) according to the manufacturer’s protocol. The previously isolated RNA was treated with DNase (Jena Bioscience, Jena, Germany).

### 2.9. Western Blotting

The electrophoretic separation of proteins from the lysate of transfected HEK293 cells was performed using SDS-PAGE according to the Laemmli method under reducing conditions in a Mini-PROTEAN vertical chamber (BioRad, Hercules, CA, USA) at a constant voltage of 130 V. Following the separation of the proteins by SDS-PAGE, they were transferred to a nitrocellulose membrane at 100 mA for 1 h for the subsequent Western blotting procedure.

The Western blotting procedure was initiated by applying 5 mL of blocking buffer (phosphate-buffered saline (PBS) containing 0.05% Tween 20 (PBST) with 1% bovine serum albumin (BSA)) to the membrane surface, followed by the addition of 25 μL of the primary antibody 29F2 (monoclonal antibody 29F2, which recognizes the marker Gag epitope EPFRDYVDRFYKTLR, kindly provided by V. A. Poryvaeva). The secondary antibodies employed in this study were goat anti-mouse IgG antibodies conjugated to horseradish peroxidase (Goat Anti-Mouse IgG Antibody, (H+L) HRP conjugate, Sigma-Aldrich, St. Louis, MO, USA). The primary and secondary antibodies were used at a dilution of 1:5000. The antibodies were incubated with the membrane for 30 min at room temperature. The immune complex was subsequently visualized through the use of the ECL Select Western Blotting Detection Reagent (GE Healthcare Life Sciences, Uppsala, Sweden). The result was visualized and analyzed using the GE AI600 Imager (GE Healthcare Life Sciences, Uppsala, Sweden).

### 2.10. Immunization of BALB/c Mice

The work with animals was carried out in accordance with the “Guide for the Care and Use of Laboratory Animals”. The protocols were approved by the Institutional Animal Care and Use Committee at the FBIS SSC VB «Vector» Rospotrebnadzor (Bioethics Committee Protocol No. 3 dated 29 February 2024). The mice were housed under a 12 h light/dark cycle with free access to food and water.

The evaluation of the immunogenic properties of the experimental DNA vaccine was conducted on inbred BALB/c mice, with an initial weight range of 16–18 g. The experiment involved a total of 36 female mice, which were obtained from the animal facility of the Institute of Cytology and Genetics of the Siberian Branch of the Russian Academy of Sciences (Novosibirsk, Russia). The animals were divided into six groups, with each group consisting of six animals. The mice were immunized on days 0 and 21. The first, second, and third groups received intramuscular (IM) injections of 100 μg of plasmid pVAX-AG1-ub, pVAX-AG2-ub, or pVAX-AG4-ub, respectively, in 50 μL of PBS, followed by electroporation; the fourth group received 100 μg of pVAX1 in 50 μL of PBS, followed by electroporation; the fifth group received an intraperitoneal injection of 0.5 mL of the Tick-E-Vac vaccine (Chumakov FSC for Research and Development of Immune-and-Biological Products of Russian Academy of Sciences (Institute of Poliomyelitis), Moscow, Russia); and the sixth group consisted of naive animals.

To immobilize the animals, inhalation anesthesia (RWD Life Science, Shenzhen, China) with a 2.5% isoflurane solution was used. The mice were initially placed in an induction chamber and subsequently transferred to anesthesia masks. The removal of the fur from the paw was performed using a depilatory gel, and the skin was treated with 70% ethanol. The plasmid was administered IM using insulin syringes with 29 G needles, followed by electroporation. Electroporation was performed using a CUY21 EDIT II electroporator and LF650P5 tweezers-type electrodes with a diameter of 5 mm (BEX Co., Ltd., Tokyo, Japan) according to a protocol with the following parameters: rectangular direct current of both forward and reverse polarity in three pulses, 12 V voltage with intervals of 30 ms and 950 ms, and a current limit of 45 mA.

To obtain blood serum from the experimental and control groups on day 35, blood was collected from the retroorbital sinus of the animals’ eyes. Animals were humanely euthanized by cervical dislocation, followed by spleen collection for T-cell response analysis.

### 2.11. Isolation of Splenocytes from Immunized BALB/c Mice

The spleens were sequentially homogenized using nylon cell filters with pore sizes of 70 and 40 μm (BD, Franklin Lakes, NJ, USA). Following red blood cell lysis with a lysis buffer (Sigma-Aldrich, St. Louis, MO, USA), the splenocytes were washed twice with RPMI complete medium and placed in 1 mL of RPMI medium containing 2 mM L-glutamine, gentamicin (50 μg/mL), and 10% FBS (Thermo Fisher Scientific, Waltham, MA, USA). The cell count was determined by means of a TC 20™ automated cell counter (BioRad, Hercules, CA, USA).

### 2.12. ELISpot

The T-cell response in immunized mice was assessed using ELISpot analysis with the Mouse IFN-gamma ELISpot kit (BD, Franklin Lakes, NJ, USA) according to the manufacturer’s instructions. To stimulate splenocytes, a pool of peptides (20 μg/mL of each peptide) restricted by MHC class I and MHC class II molecules and corresponding to epitopes from TBEV proteins included in the construct sequences was used (Table 1). The prediction of peptides for the designed immunogens was conducted using the NetMHCpan-4.1 and NetMHCIIpan-4.0 services based on the %Rank parameter. The selected peptides were subsequently synthesized by the commercial company “AtaGenix Laboratories” (Wuhan, China).

The number of IFN-γ-producing cells was counted using a Carl Zeiss (Oberkochen, Germany) ELISpot analyzer.

### 2.13. Enzyme-Linked Immunosorbent Assay (ELISA)

ELISA was performed using a recombinant E protein from TBEV (kindly provided by staff members of the Department of Molecular Virology of Flaviviruses and Viral Hepatitis at the FBIS SSC VB «Vector»; a detailed procedure for obtaining the protein is described in patent document RU2136754C1) as the antigen. The antigen (1 μg/mL in 2 M urea) was adsorbed onto 96-well plates (Greiner Bio-One, Kremsmünster, Austria) at 4 °C for a duration of 16 h; the plate was then washed with PBST and blocked with a 1% casein solution in the same buffer for 60 min at room temperature. Sera were subsequently introduced into the wells in three-fold serial dilutions, starting at 1:50 in the blocking solution, and then incubated for a duration of 60 min at room temperature. The plate was washed, horseradish peroxidase-conjugated rabbit anti-mouse IgG antibodies (Sigma-Aldrich, St. Louis, MO, USA) were added, and the plate was incubated for 60 min at room temperature. Then, 3,3′,5,5′-tetramethylbenzidine (TMB) (Amresco, Solon, OH, USA) was added to the wells of the plate, which had been washed with PBST. The optical density was measured at a wavelength of 450 nm using a Varioskan LUX (Thermo Fisher Scientific, Waltham, MA, USA). The serum dilution at which the optical density value was more than twice that of the negative control (in which blocking buffer was added to the wells instead of serum) was the last dilution that gave a positive response. The final titer was determined as the last diluted sample that gave positive results for ELISA.

### 2.14. Statistical Analysis

A statistical analysis of the resulting immune response data was conducted using GraphPad Prism 9.0 (GraphPad Software, Inc., San Diego, CA, USA). The assessment of intergroup differences was conducted through the use of the nonparametric Kruskal–Wallis one-way analysis of variance, with subsequent adjustment for multiple comparisons and the implementation of Dunn’s statistical hypothesis testing. The data are provided as the median with range. Statistical significance was defined as *p* < 0.05.

## 3. Results

### 3.1. Design of Multi-Epitope Immunogens

#### 3.1.1. Epitope Selection

As an initial step in our study, we conducted a thorough analysis of the experimental data concerning the immunization of mice with flaviviruses, which is available in the IEDB. Data on all known flavivirus T-cell epitopes were downloaded. A total of 1386 mouse T-cell epitopes were identified, 707 of which were MHC I-restricted and 698 of which were MHC II-restricted.

In the initial round of selection, all T-cell epitopes were aligned against the TBEV proteome. Subsequent to an overlap analysis, short protein fragments carrying the largest number of valid peptide ligands that aligned with them were identified from the proteome. Peptides with an identity level of more than 70% and with a maximum of five amino acid substitutions, insertions, or deletions were selected for further analysis. Consequently, 119 peptides were selected. Peptides with sequence overlaps were combined into larger fragments. A total of 42 oligopeptides, ranging in length from 7 to 44 amino acid residues, were selected.

This approach was chosen because it allows the vaccine to include not only the maximum number of short T-killer epitopes but also longer T-helper epitopes. The oligopeptides that were selected at this stage are provided in Table 2.

In the second round of selection, binding affinity predictions for MHC alleles of the BALB/c mouse strain were obtained for all 9-residue peptides isolated from the oligopeptides. These predictions were made using the NetMHCpan 4.1 software program. The selection criterion required the inclusion of peptides for which at least one epitope was predicted to be restricted by H-2-Kd, H-2-Dd, or H-2-Ld. Furthermore, the whole T-helper epitopes were evaluated using NetMHCIIpan 4.0. To this end, binding affinity predictions for H-2-IAd and H-2-IEd alleles were obtained. Consequently, 13 oligopeptides were selected.

The final epitopes were selected based on their ability to bind to different MHC alleles, as well as on general criteria for peptide design [33]. The epitopes selected at this stage are provided in Table 3. Consequently, three peptides were isolated from the envelope protein (E), one from nonstructural protein 1 (NS1), one from the serine protease (NS3), and eight from the RNA-dependent RNA polymerase (NS5).

#### 3.1.2. Design of Multi-Epitope Constructs

**Figure 1:**
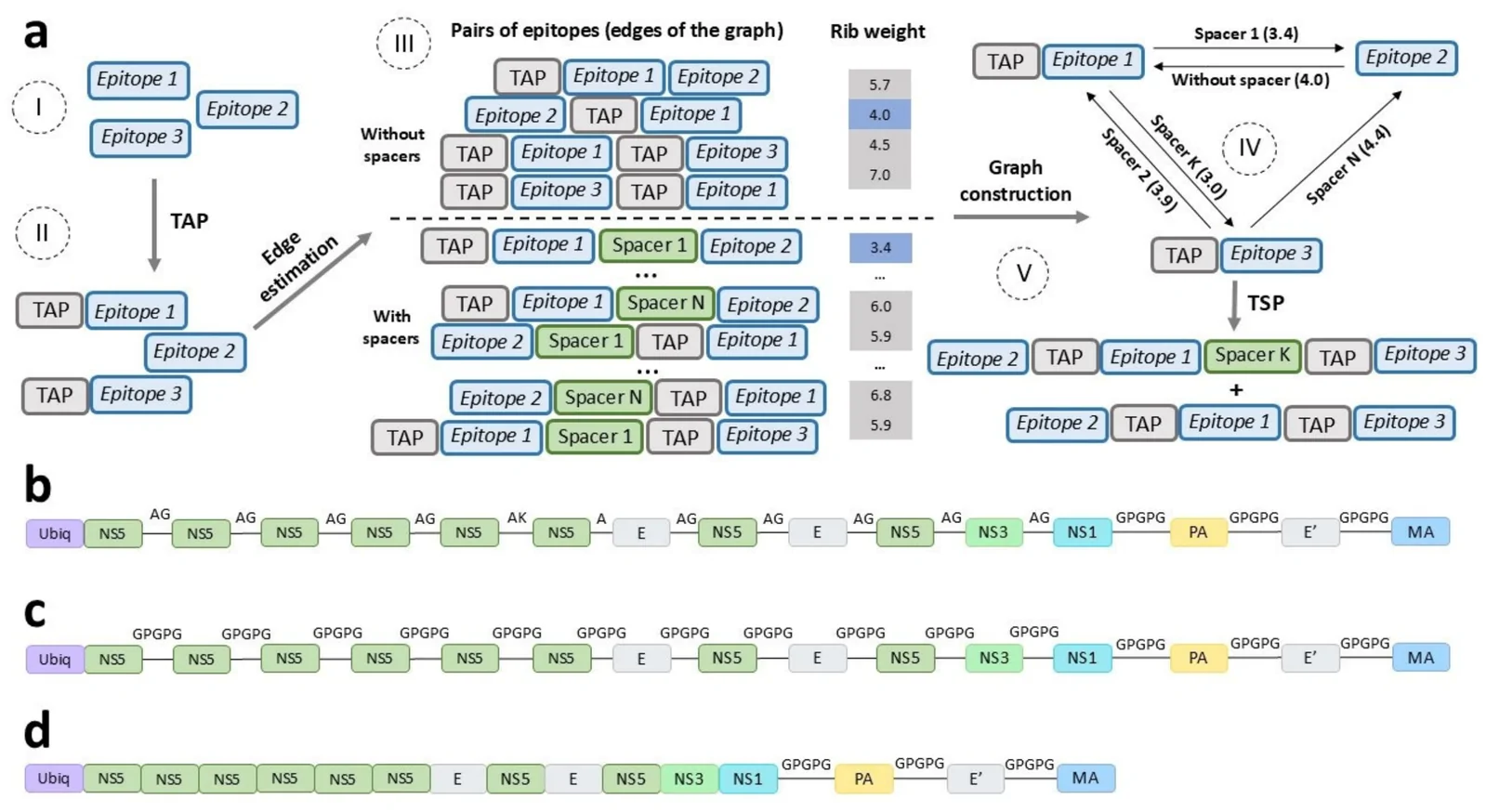
Algorithm for designing multi-epitope immunogens. (**a**) Flowchart of PolyCTLDesigner (described in detail at http://tepredict.sourceforge.net/PolyCTLDesigner.html (accessed on 10 April 2023)): I. Selection of T-cell epitopes. II. Prediction of peptide affinity for TAP. III. Predicting optimal spacer sequences for each peptide pair and evaluating them based on proteasomal cleavage efficiency, spacer length, number of predicted off-target epitopes at the junction, and epitope rank. IV. Construction of a weighted graph in which the nodes are target epitopes and the edges represent possible combinations of them. V. Solve the traveling salesman problem and design target sequences. Schematic representation of multi-epitope T-cell immunogens AG1-ub (**b**), AG2-ub (**c**), and AG4-ub (**d**).

The following strategies (Figure 1) were chosen for the final assembly of the multi-epitope vaccine from the selected epitopes:

1. The oligopeptides were linked using spacer sequences selected with the PolyCTLDesigner program.
2. The oligopeptides were linked using a GPGPG spacer.
3. The oligopeptides were linked without using spacer sequences.

PolyCTLDesigner was used to determine the order of the oligopeptides in the constructs. Then, spacer sequences were selected and incorporated into the final constructs.

To optimize the selection of spacers for the first construct (AG1-ub), PolyCTLDesigner, which employs graph-based algorithms to solve this problem, was used. We used a degenerate sequence [A][KHR] as a spacer to test all combinations of amino acids and identify the optimal amino acid motif for each pair of epitopes that minimizes the negative effects of their interaction. This sequence was chosen because the presence of alanine at the N-terminus of the spacer improves processing by immunoproteasomes [15,34], and a positively charged amino acid at the second position increases binding affinity to human TAP [35].

The second strategy for assembling a multi-epitope immunogen (AG2-ub) used the aforementioned GPGPG spacers to link the peptide fragments. The third immunogen (AG4-ub) was designed with the same oligopeptides but without spacer sequences.

Ubiquitin was added to the N-terminus of all constructs. The EPFRDYVDRFYKTLR marker epitope, which is recognized by the 29F2 monoclonal antibody, and the universal T-cell epitope PADRE (AKFVAAWTLKAAA) were added to the C-terminus. The complete amino acid sequences of the developed immunogens are provided in Figure S1.

**Figure 2:**
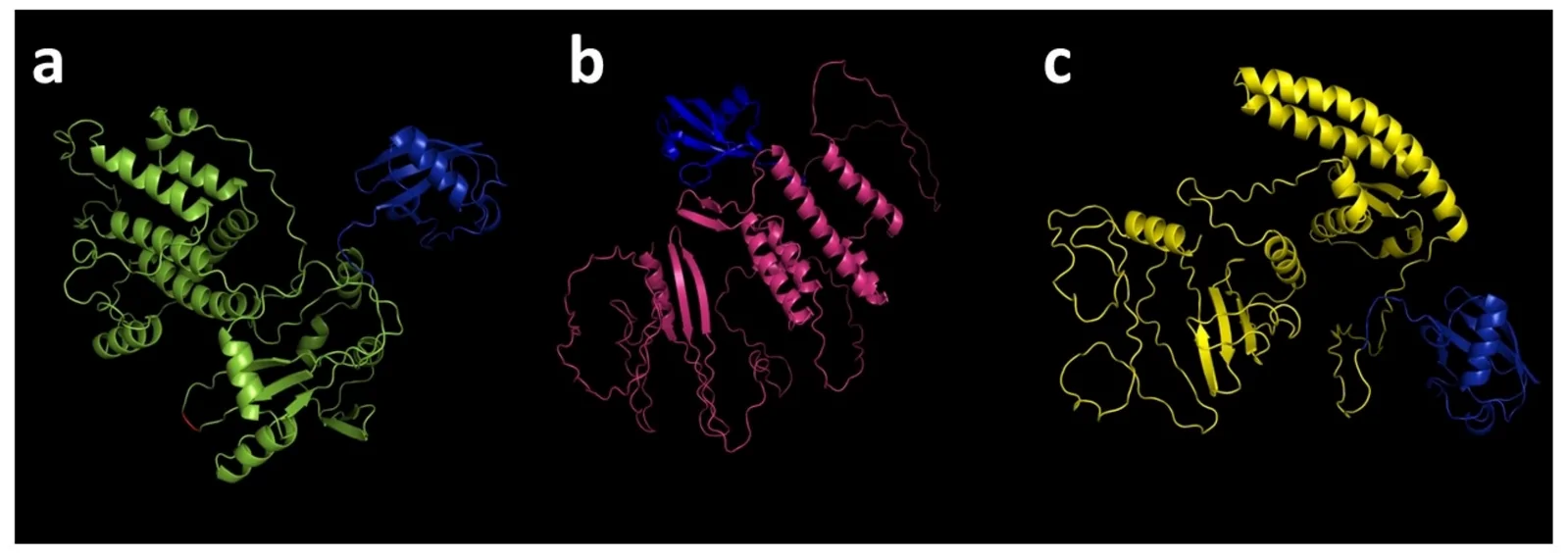
Hypothetical 3D structures of artificial T-cell immunogens. Structural models predicted for (**a**) AG1-ub; (**b**) AG2-ub; (**c**) AG4-ub (obtained using the AlphaFold Server). The figure shows the main part of the immunogen in green, pink, and yellow. The ubiquitin fragments attached to the immunogens are shown in blue.

Despite the amino acid sequence differences among the immunogens AG1-ub, AG2-ub, and AG4-ub, a three-dimensional (3D) structure analysis using the AlphaFold Server did not reveal significant variations among them (Figure 2).

A subsequent analysis of the secondary structure of the proteins using the PSIPRED algorithm revealed that the percentage of alpha-helices, beta-sheets and unstructured elements in the predicted structures did not differ significantly (Table 4).

The predicted physicochemical properties of the AG1-ub, AG2-ub, and AG4-ub proteins also did not differ significantly (Table 5).

### 3.2. Production of DNA Plasmids Encoding Multi-Epitope T-Cell Immunogens

The nucleotide sequences of the AG1-ub, AG2-ub, and AG4-ub immunogens were obtained. The obtained sequences were then subjected to codon optimization and subsequent synthesis (Figure S2). Thereafter, three experimental DNA vaccine constructs were generated based on the pVAX1 plasmid vector: pVAX-AG1-ub, pVAX-AG2-ub, and pVAX-AG4-ub.

### 3.3. Analysis of the Target Gene Expression Using RT-PCR and Western Blot

**Figure 3:**
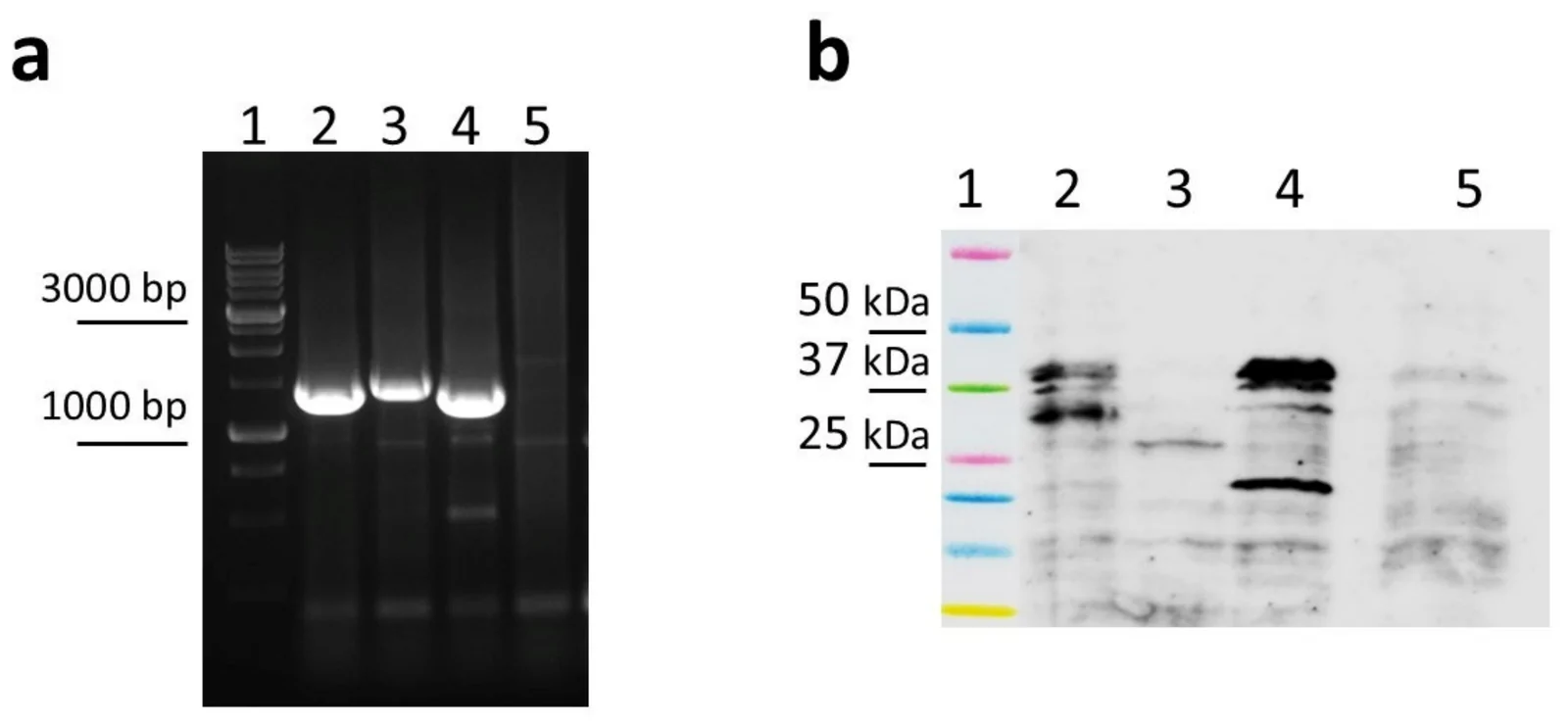
Evaluation of the ability of DNA vaccine constructs to express target genes in eukaryotic cells. (**a**) Electrophoretic analysis of RT-PCR products on a 1% agarose gel: lane 1—M12 molecular weight marker (SibEnzyme, Novosibirsk, Russia); lanes 2, 3, 4—AG1-ub, AG2-ub and AG4-ub PCR products obtained using cDNA as a template and specific primers; lane 5—PCR products obtained using specific primers and total RNA isolated from untransfected HEK293 cells. (**b**) Analysis of ag1-ub, ag2-ub and ag4-ub gene expression in HEK293 cells transfected with pVAX-AG1-ub, pVAX-AG2-ub and pVAX-AG4-ub by Western blotting: lane 1—marker; lanes 2, 3, 4—cell lysates from cells transfected with pVAX-AG1-ub, pVAX-AG2-ub and pVAX-AG4-ub, respectively; lane 5—lysate from untransfected cells.

The ability of DNA constructs to mediate the expression of a target gene in eukaryotic cells was assessed based on the synthesis of specific mRNA. The synthesis of mRNA of the theoretically calculated size was observed in eukaryotic cells for all of the resulting artificial genes encoding the AG1-ub, AG2-ub, and AG4-ub immunogens, as confirmed by RT-PCR. The results are displayed in the electrophoregram (Figure 3a). The whole electrophoregram is shown in Figure S3.

Western blotting revealed that the transfected cells contained the AG1-ub, AG2-ub, and AG4-ub proteins, as well as their processing products. As demonstrated in Figure 3b, the AG1-ub and AG4-ub proteins are present in two forms. The first form consists of full-length proteins without ubiquitin and corresponds to the theoretically calculated molecular weights of the target protein product in the absence of ubiquitin: AG1-ub—39 kDa and AG4-ub—37.5 kDa (molecular weights of the full-length proteins: AG1-ub—47.6 kDa and AG4-ub—46.1 kDa). It appears that ubiquitin is removed from proteins by the proteasome during processing with relatively high efficiency. The second form consists of a set of discrete bands. This observation suggests the possibility of specific processing of these proteins in eukaryotic cells. The AG2-ub protein was identified as the one undergoing the most processing. It was detected exclusively in its processed form, with the predominant band located at approximately 30 kDa, which is about half of the theoretically calculated molecular weight (50.2 kDa). The whole image of Western blotting is shown in Figure S4.

The results of the densitometric analysis (File S1) demonstrate that the total amount of AG1-ub and AG4-ub protein products exceeds that of AG2-ub. However, it cannot be definitively stated that these results are due to lower expression of the AG2-ub protein compared to the other constructs, since the specific characteristics of the constructs and the rate at which they are processed preclude any comparison of the expression of full-length, unprocessed protein products without conducting additional studies.

### 3.4. Evaluation of the Immunogenic Properties of Experimental DNA Vaccine Constructs

To evaluate the immunogenicity of experimental DNA vaccine constructs, three groups of animals were immunized with pVAX-AG1-ub, pVAX-AG2-ub, or pVAX-AG4-ub IM followed by electroporation. Two groups of animals served as negative controls: one group was immunized with the pVAX1 plasmid vector, and the other group consisted of naive animals. The group of animals that received the commercial vaccine served as the positive control. The mice were immunized twice, on days 0 and 21.

**Figure 4:**
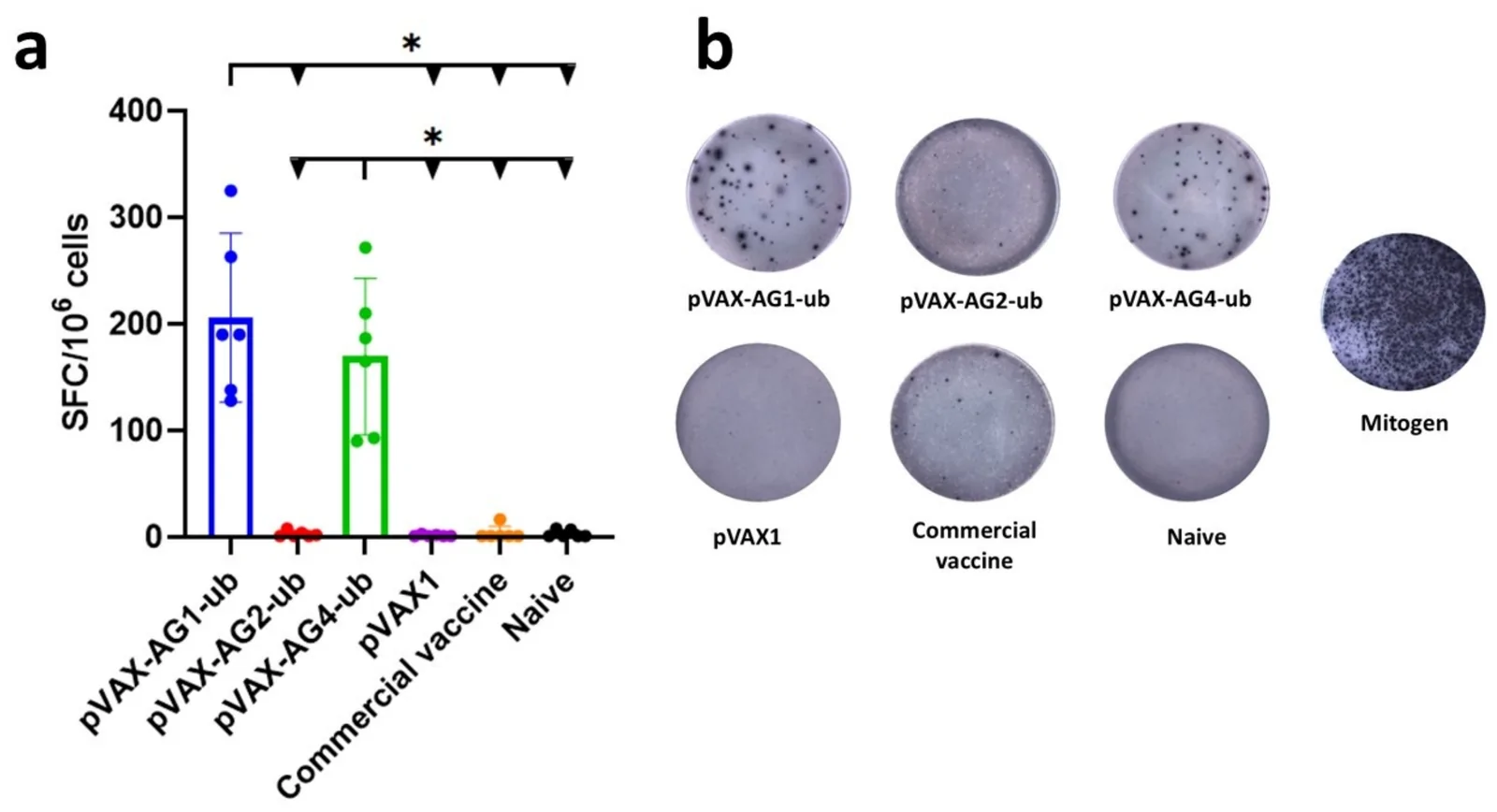
Results of the analysis of the cellular immune response in BALB/c mice (number of animals: *n* = 6). (**a**) Number of splenocytes expressing IFN-γ in response to stimulation with a panel of specific peptides, as determined by the ELISpot assay. Each bar represents the mean number of spot-forming cells (SFC) per million stimulated splenocytes. Data are provided as the median with range. The dots on the graph represent individual data points. Statistical significance was determined using a nonparametric Kruskal–Wallis analysis of variance with correction for multiple comparisons and a Dunn’s post hoc test (* *p* < 0.05) (File S2). (**b**) Representative images of ELISpot wells.

Fourteen days after the second immunization, spleens were collected from six animals in each group. The immune response was assessed using ELISpot analysis. The analysis revealed that the highest number of splenocytes producing IFN-γ in response to stimulation with a pool of TBEV protein-derived peptides was observed in groups of mice immunized with pVAX-AG1-ub and pVAX-AG4-ub (Figure 4). However, the level of the T-cell response in the pVAX-AG1-ub group was not statistically significantly higher than in the pVAX-AG4-ub group. The cellular response was at background levels in the control groups of animals immunized with the Tick-E-Vac and pVAX1 vaccines.

**Figure 5:**
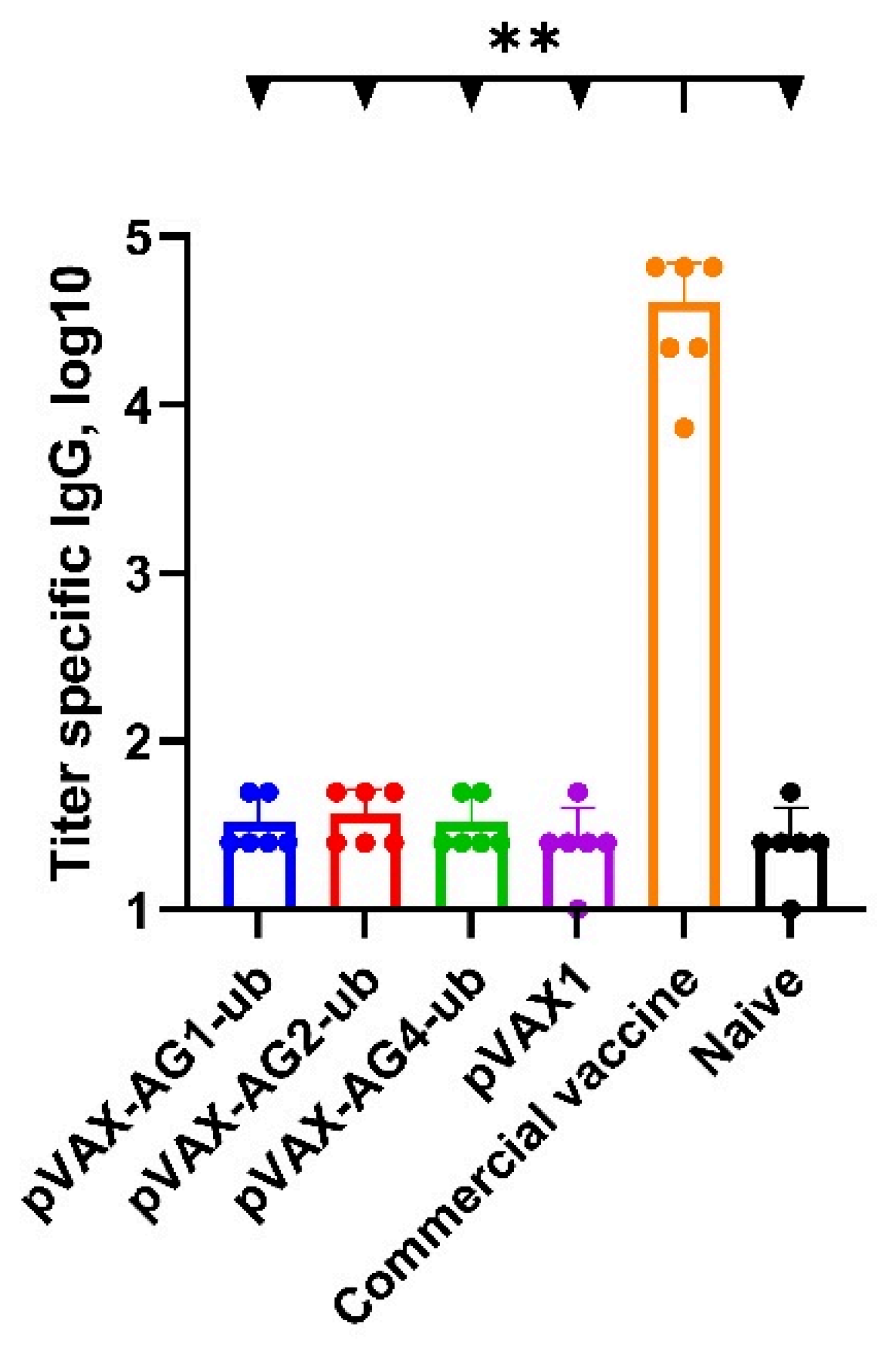
Results of the analysis of the humoral immune response in BALB/c mice (number of animals: *n* = 6). Reverse titer (dilution) of specific antibodies against the TBEV E protein detected in immune sera by ELISA. Data are provided as the median with a range. The dots on the graph represent individual data points. Statistical significance was determined using a nonparametric Kruskal–Wallis analysis of variance with correction for multiple comparisons and a Dunn’s post hoc test (** *p* < 0.01) (File S3).

ELISAs of individual mouse blood sera were performed 14 days after the second immunization. The results (Figure 5) showed a high antibody titer in the sera of mice immunized with the commercial vaccine but not in those from the pVAX-AG1-ub, pVAX-AG2-ub, pVAX-AG4-ub, or pVAX1 groups.

This result is consistent with our predictions and can be explained by the fact that the multi-epitope DNA constructs contain only T-cell epitopes from the nonstructural proteins NS1, NS3, and NS5, as well as the E protein. These constructs lack the B cell epitopes that are responsible for the formation of the humoral immune response. Additionally, we used the recombinant TBEV E protein as the antigen in the ELISA. Consequently, we did not detect specific antibodies in the sera of immunized animals.

## 4. Discussion

There is increasing scientific interest in artificial multi-epitope T-cell immunogens, as evidenced by the rising number of publications on this subject [36,37]. A search on ClinicalTrials.gov using terms such as “multi-epitope vaccine” and “polyepitope vaccine” reveals more than a hundred different studies that have entered clinical trials.

DNA and mRNA vaccines are frequently employed in the development of vaccine constructs based on multi-epitope immunogens, given their ability to encode complex artificial genes and to ensure their expression in eukaryotic cells.

As previously mentioned, there is currently no single “algorithm” for developing multi-epitope vaccines. In this study, we used TBEV as an example to propose several strategies for designing multi-epitope DNA vaccine constructs. We then evaluated the influence of significant theoretical factors, such as epitope selection, spacer selection, and spacer arrangement, on immunogenicity through experimentation.

The efficacy of a T-cell immunogen depends on the selection of epitopes that ensure a sufficiently high level of immunogenicity [38,39]. Guided by this principle, we used open-access data from the IEDB 2.22, as well as a combination of NetMHCpan-4.1 and NetMHCIIpan-4.0, to more accurately predict T-cell epitopes in the TBEV polyprotein. Since selecting both immunodominant and subdominant epitopes is important for ensuring a broad repertoire of T-cell responses in the event of a viral genome mutation [38], we focused on selecting conservative fragments (oligopeptides) containing a large number of epitopes from the NS1, NS3, NS5, and E proteins. These fragments formed the basis for all three designed multi-epitope antigen constructs.

Another important factor is how efficiently the peptides that compose the vaccine are processed and presented [15,38,39]. This may be influenced by the spacer sequences surrounding the epitopes within the construct.

In the present study, two types of spacers were used: GPGPG and alanine-containing spacers based on [A][KHR]. Each of these has its own theoretical rationale. GPGPG is a glycine-rich flexible spacer that is frequently selected for the purpose of connecting T-helper epitopes in multi-epitope constructs. Its advantages include high flexibility, which allows adjacent epitopes to move independently and maintain the correct conformation, as well as a reduced risk of forming undesirable “junctional epitopes” at the junctions between epitopes.

Currently, researchers developing multi-epitope vaccines designed to activate the T-cell component of the immune response most commonly choose rigid spacers containing alanine, tyrosine, and leucine (e.g., (AAY) or (EAAAK)n). These spacers are preferred cleavage sites for the immunoproteasome and the ERAP1/2 complex; cleavage at these sites contributes to effective processing and presentation via the MHC class I pathway [15,40,41].

This approach significantly simplifies the design process by allowing the use of effective spacer options without additional research. However, it has disadvantages. For example, the inclusion of certain spacers has been shown to lead to the emergence of undesirable neoepitopes. Therefore, their use must be verified when designing a multi-epitope construct [42]. Furthermore, a study on the theoretical modeling of optimal spacer sequences showed that selecting spacers on a case-by-case approach for each multi-epitope construct increases the probability of correct and efficient antigen processing [39].

The amino acids surrounding the peptide may also significantly impact this. Therefore, when discussing the importance of peptide positioning in vaccine design, we are referring specifically to the optimal flanking amino acid sequences for each pair of epitopes [38,39].

Based on the above, three multi-epitope antigen constructs were designed using various strategies to optimize immunogenicity, specifically different strategies to select spacer sequences. To address the issue of epitope arrangement within the constructs, we used PolyCTLDesigner, which ensures an optimal epitope order for processing and subsequent presentation.

For the first multi-epitope variant, we chose the classic peptide-linking approach that uses alanine-containing spacer sequences. In our case, we used the degenerate sequence [A][KHR]. Using PolyCTLDesigner, we identified spacers for each pair of epitopes that could potentially maximize the presentation of each epitope (AG1-ub). To evaluate the effectiveness of this approach, two additional constructs comprising the same oligopeptides but differing in linking were developed for comparison. The spacers were removed from the AG4-ub multi-epitope construct, whereas flexible GPGPG spacers were used instead of optimally selected sequences for the AG2-ub construct.

Subsequently, three experimental DNA vaccines, designated pVAX-AG1-ub, pVAX-AG2-ub, and pVAX-AG4-ub, encoding TBEV multi-epitope immunogens, were produced. The expression of the target genes was confirmed using RT-PCR and Western blotting.

The immunogenicity of the designed multi-epitope immunogens was investigated by immunizing mice with DNA vaccines via electroporation. Antibodies against TBEV structural proteins were detected exclusively in mice immunized with the commercial vaccine. Of the three DNA vaccines examined, none induced a significant antibody response against TBEV proteins in the animals (Figure 5). These results are consistent with the predictions made and also indicate that the selection of T-cell epitopes within the construct was performed correctly.

Concurrently, ELISpot analysis revealed that the pVAX-AG1-ub and pVAX-AG4-ub DNA vaccines effectively induced a virus-specific T-cell response, in contrast to the commercial vaccine. Although the T-cell response level in the pVAX-AG1-ub group was not statistically significantly higher than in the pVAX-AG4-ub group, there was a trend toward increased immunogenicity of the construct with optimized spacers (AG1-ub). It can be hypothesized that the inclusion of alanine spacers in the AG1-ub construct may enhance its immunogenicity compared to the AG4-ub construct, apparently due to improved processing and presentation of epitopes, as has already been demonstrated in a number of studies [17,43,44].

These results suggest that an optimal epitope sequence without properly selected flanking sequences does not fully realize the immunogen’s potential. Optimally selected spacers with a length of three or more amino acid residues may contribute to a greater increase in the immunogenicity of the DNA vaccine. The following experiments will test this hypothesis. Meanwhile, the pVAX-AG2-ub construct with GPGPG flexible spacers demonstrated a low immunogenicity profile, as evidenced by its inability to elicit a cytotoxic response.

In order to explain the low immunogenicity of AG2-ub, two hypotheses have been formulated. One hypothesis suggests that an overly flexible spacer could have led to the instability of the entire molecule and, possibly, to its subsequent aggregation, which could result in the protein being directed to the lysosomal pathway [45,46,47]. As illustrated in Figure 3b, the protein profile resulting from AG2-ub processing exhibits significant differences compared to those derived from AG1-ub and AG4-ub protein processing. This finding may suggest the presence of an alternative degradation pathway for AG2-ub, though further investigation is necessary to substantiate this hypothesis. The second hypothesis is related to the characteristics of the proteasomal proteolysis mechanism. The preferred cleavage sites for the proteasome are regions following hydrophobic or basic amino acids [44]. The incorporation of glycine- and proline-rich spacers (GPGPG) has been demonstrated to impede the optimal functionality of proteases and protein processing, thereby influencing the protein’s immunogenicity.

The data obtained confirm the notion that the choice of spacer cannot be arbitrary and that GPGPG proved ineffective in this case. At the same time, some other studies focused on the development of multi-epitope vaccines—particularly those designed to activate a T-helper response—have shown opposite results [48,49]. A study by Abdullah et al. [50] showed that the incorporation of flexible glycine-based spacers (GGGS and GPGPG) within a multi-epitope vaccine designed to combat leptospirosis exhibited notable efficacy in stimulating a balanced Th1/Th2 cellular response. A study of the immunogenic properties of DNA vaccines encoding multi-epitope HIV immunogens by Yang et al. [16] yielded even more contradictory data. A comparative analysis of multi-epitope vaccines comprising the common spacer sequences GGGS or AAY revealed that the design incorporating flexible spacers exhibited enhanced immunogenicity, including a CTL response.

Thus, obtaining new experimental data on the development of multi-epitope immunogens is crucial for developing modern therapeutic strategies that aim to prevent various diseases and improve quality of life.

It is imperative to acknowledge the limitations of this study when interpreting the results. Firstly, despite the detection of AG2-ub processing products, it remains unconfirmed that the low immunogenicity of this construct is attributable to protein instability or degradation. This issue necessitates further investigation, for example, through the use of protease inhibitors. Secondly, it should be noted that the conclusions of this study are applicable exclusively to the BALB/c mouse model (H-2^d^ haplotype). It is possible that the immunogenicity of these constructs in mice with different genetic variants or in humans may differ. Thirdly, for the AG1-ub immunogen, the PolyCTLDesigner algorithm was employed to select alanine-containing spacers of a relatively small size. However, the extant literature suggests that when selecting spacers containing a greater number of amino acid residues (three or more), the immunogenicity of such constructs may increase significantly. In the future, larger-scale studies will be required to address these limitations and further validate our approach.

## 5. Conclusions

In this study, a comparative analysis was conducted on three approaches to combining TBEV protein T-cell epitopes in an artificial immunogen: the use of inter-epitope spacers (optimized alanine-containing spacers or flexible GPGPG spacers) and the absence of spacers. It was demonstrated that the choice of spacer significantly affects the immunogenicity of multi-epitope T-cell DNA vaccines. The spacer-free construct (AG4-ub) and the construct with alanine spacers that was optimized using PolyCTLDesigner (AG1-ub) elicited a significant virus-specific T-cell response in BALB/c mice, as demonstrated by IFN-γ-ELISpot. Meanwhile, the construct with the GPGPG spacer (AG2-ub) exhibited low immunogenicity, likely due to impaired protein expression or processing. None of the DNA vaccines induced antibody production against TBEV structural proteins, confirming their T-cell-directed activity. While the difference between AG1-ub and AG4-ub did not reach statistical significance, the observed trend supports the hypothesis that individually optimized spacers can enhance antigen processing and presentation. These results provide the first experimental evidence of the importance of spacer selection in developing a multi-epitope vaccine against TBEV. They also confirm the effectiveness of computational tools, such as PolyCTLDesigner. Further research is needed to optimize these vaccines and evaluate their protective efficacy.

## Figures and Tables

**Table 1:** Peptides used for the specific stimulation of splenocytes.

Amino Acid Sequence of the Peptide	Purity, %
LSYFHRRDL	≥80
LVMKDGRTL	≥80
RDWFNDLAL	≥80
STTESGKVI	≥80
HEMYYSTAV	≥80
SPTMGPATL	≥80
STAVTGNIV	≥80
LTNIKVQLI	≥80
YMWLGSRFL	≥80
LAINSAVPV	≥80
FSRNSTHEMYYSTAVTGN	≥80
GASVRSTTESGKVIPEWSSRASTMP	≥80
GRGGWSYYAASRPAV	≥80
HELVMKDGRTLVVPSRDQDEL	≥80
SKGVLHTMWHVTRGAALS	≥80
STAVTGNIVNSVNVQSRKLLARF	≥80
VHRDWFNDLALP	≥80
DRGWGNHSGLFGKG	≥80

**Table 2:** Selected oligopeptides.

Peptides	Length	Location Within the TBEV Polyprotein
SPTMGPATL	9	E
VHRDWFNDLALP	12	E
DRGWGNHSGLFGKG	14	E
VTLVLELGGCVTITA	15	E
KRDQSDRGWGNHCGLFGKGSIVACVKAACEA	31	E
KPCRIPVRAVA	11	E
WDFGSAGGFLSSIGKALH	18	E
GASVRSTTESGKVIPEWSSRASTMP	25	NS1
SKGVLHTMWHVTRGAALS	18	NS3
DVREDVV	7	NS3
AIPIDLVKGTSGSPILNAQGVVVGLYGNGL	30	NS3
CIDRRLRTLVLAPTRVVLKEMERALNG	27	NS3
VAIMDEAHWTDPHSIAARGHLY	22	NS3
LVLMTATPPGKSEPFPESNGAI	22	NS3
PDFVVTTDISEMGANLDVSRVIDGR	25	NS3
RVTTASAAQRRGRVGR	16	NS3
FGDVLTGM	8	NS4A
LASLLLLWA	9	NS4A
ALIFYTLL	8	NS4A
LMVGVGLAA	9	NS4B
FSRNSTHEMYYSTAVTGN	18	NS5
GRGGWSYYAASRPAV	15	NS5
STAVTGNIVNSVNVQSRKLLARF	23	NS5
GSIMDVITRRDQRGSGGQVVTYALNTLTNIKVQLIRMMEGEGVI	44	NS5
HELVMKDGRTLVVPSRDQDEL	21	NS5
WSVRETASLSKAYGQMWLLSYFHRRDLRTLGLAINSAVPV	40	NS5
PWNAREDVVRMAMTDTTAFGQQRVFK	26	NS5
EFGVAKGSRAIWYMWLGSRFLEFEALGFLNE	31	NS5
TLGDLWKRRLNNCTREEF	18	NS5
LAVSRGTAKL	10	NS5
MCDIGESSPDAAVEGERT	18	NS5
VLAPYRPEV	9	NS5
FSRNSTHEMYY	11	NS5
GSYRTAPTGSAASLINGVVKLL	22	NS5
KPRMCSREEFIAKVKSNAALG	21	NS5
ERHLMGRCAHCVYNMMGKREKKLG	24	NS5
DHWASRESSGAGVEGISL	18	NS5
TLNGGLFYADDTAGWDT	17	NS5

**Table 3:** Selected oligopeptides.

Peptides	Length	Location Within the TBEV Polyprotein	Location Within the TBEV Protein Domain
SPTMGPATL	9	E	Сentral/dimerization domains
VHRDWFNDLALP	12	E	Сentral/dimerization domains
DRGWGNHSGLFGKG	14	E	Сentral/dimerization domains
GASVRSTTESGKVIPEWSSRASTMP	25	NS1	β-ladder domain
SKGVLHTMWHVTRGAALS	18	NS3	N-terminal serine protease domain
GRGGWSYYAASRPAV	15	NS5	N-terminal methyltransferase (MTase) domain
STAVTGNIVNSVNVQSRKLLARF	23	NS5	N-terminal methyltransferase (MTase) domain
GSIMDVITRRDQRGSGGQVVTYALNTLTNIKVQLIRMMEGEGVI	44	NS5	RNA-dependent RNA polymerase (RdRp) catalytic domain
HELVMKDGRTLVVPSRDQDEL	21	NS5	RNA-dependent RNA polymerase (RdRp) catalytic domain
WSVRETASLSKAYGQMWLLSYFHRRDLRTLGLAINSAVPV	40	NS5	RNA-dependent RNA polymerase (RdRp) catalytic domain
PWNAREDVVRMAMTDTTAFGQQRVFK	26	NS5	RNA-dependent RNA polymerase (RdRp) catalytic domain
EFGVAKGSRAIWYMWLGSRFLEFEALGFLNE	31	NS5	RNA-dependent RNA polymerase (RdRp) catalytic domain
FSRNSTHEMYYSTAVTGN	18	NS5	N-terminal methyltransferase (MTase) domain

**Table 4:** The predicted secondary structure of the proteins using the PSIPRED algorithm.

Immunogen	Percentage, %		
	Alpha-Helices	Beta-Sheets	Unstructured Elements
AG1-ub	33	17	50
AG2-ub	30	14	56
AG4-ub	34	11	55

**Table 5:** The predicted physicochemical properties of the multi-epitope immunogens.

Immunogen	Value of a Quantity		
	pI	GRAVY	Instability Index
AG1-ub	9.90	−0.267	33.74
AG2-ub	9.87	−0.376	31.02
AG4-ub	9.87	−0.310	35.40

## Data Availability

The data can be shared upon request.

## Supplementary Materials

The following supporting information can be downloaded at: https://www.mdpi.com/article/10.3390/vaccines14090770/s1, Figure S1: Amino acid sequences of designed immunogens; Figure S2: Nucleotide sequences of designed immunogens; Figure S3: The whole electrophoregram of PCR analysis; Figure S4: The whole image of Western blot analysis; File S1: Densitometry of Western blot analysis; File S2: Kruskal–Wallis test ELISpot with Dunn’s multiple comparisons test; File S3: Kruskal–Wallis test ELISA with Dunn’s multiple comparisons test.

## Abbreviations

The following abbreviations are used in this manuscript:

TBEV	Tick-borne encephalitis virus
DNA	Deoxyribonucleic acid
IEDB	Immune Epitope Database
CTL	Cytotoxic T lymphocyte
HTL	Helper T lymphocyte
MHC	Major histocompatibility complex
%Rank	Percentile rank
pI	Isoelectric point
GRAVY	Grand average of hydropathy
PSIPRED	Protein Structure Prediction Server
FBS	Fetal bovine serum
RT-PCR	Reverse transcription polymerase chain reaction
cDNA	Complementary DNA
RNA	Ribonucleic acid
mRNA	Messenger RNA
PBS	Phosphate-buffered saline
PBST	PBS containing 0.05% Tween 20
BSA	Bovine serum albumin
IM	Intramuscular
ELISA	Enzyme-linked immunosorbent assay
TMB	Tetramethylbenzidine
TAP	Transporter associated with antigen processing
2D	Two-dimensional
3D	Three-dimensional
