# Supplementary material for: How Does Inter-Epitope Spacer Variation Within Artificial Immunogens Based on T-Cell Epitopes of Tick-Borne Encephalitis Virus Affect Immunogenicity?
Source: Vaccines (Basel). 2026 Sep 2;14(9):770. doi: 10.3390/vaccines14090770 (PMC13611838; doi:10.3390/vaccines14090770)
Supplement: Supplementary file 1 [file vaccines-14-00770-s001.zip › Supplementary Material Figure S2. Nucleotide sequences of designed immunogens.pdf]

Figure S2. Nucleotide sequences of designed immunogens.

ag1-ub

ATGCAAATCTTTGTAAAAACCCTGACCGGAAAGACCATCACCTTGGAAGTGGAACCTAGTGACACTATTG
AAAATGTTAAGGCTAAGATACAGGATAAAGAGGGGATCCCTCCTGACCAGCAACGGCTCATCTTTGCTGG
TAAACAGCTCGAGGACGGACGGACTCTGTCTGATTACAATATCCAGAAGGAAAGCACCCTACACCTGGTT
CTAAGACTGCGAGGTGTACGTACGTTCTCAAGAAATTCAACTCACGAGATGTATTACAGTACCGCCGTGA
CAGGCAACGCCGGAGGACGCGGCGGATGGTCTTACTATGCCGCAAGCCGGCCTGCTGTGGCCGGGTCTAC
CGCTGTTACAGGCAATATCGTGAATAGTGTGAATGTCCAGTCACGAAAACTGCTCGCCCGATTTGCGGGG
GGCTCTATCATGGACGTTATCACTCGGAGGGACCAGAGGGGCAGCGGTGGACAGGTAGTGACATACGCTT
TAAACACGCTGACTAATATCAAGGTCCAGTTGATTCGGATGATGGAAGGCGAAGGAGTGATCGCTGGTCA
CGAGCTGGTAATGAAGGATGGAAGAACCCTTGTGGTCCCCTCAAGAGACCAGGACGAGCTCGCCAAATGG
TCAGTACGCGAAACCGCCAGCTTGTCAAAGGCCTACGGACAGATGTGGCTACTGTCTTACTTCCACCGCC
GGGATCTCCGGACTCTCGGACTGGCAATCAATTCCGCTGTACCTGTTGCTTCGCCTACGATGGGTCCCGC
CACCCTGGCAGGCCCCTGGAACGCACGGGAGGACGTTGTTCGGATGGCCATGACGGACACAACCGCATTT
GGGCAGCAGAGGGTGTTCAAAGCCGGAGTCCACAGAGATTGGTTTAACGACCTCGCCCTCCCAGCTGGCG
AATTTGGAGTTGCTAAAGGATCTAGGGCGATTTGGTACATGTGGTTGGGCAGCCGTTTCCTGGAGTTTGA
GGCTTTGGGTTTCCTGAACGAGGCGGGTAGTAAGGGTGTCTTGCATACAATGTGGCATGTAACCCGCGGA
GCCGCCCTAAGCGCCGGCGGAGCAAGCGTTAGGTCTACCACAGAAAGCGGCAAAGTCATCCCGGAATGGA
GCTCACGTGCATCTACTATGCCAGGGCCAGGACCTGGCGCTAAGTTCGTTGCAGCCTGGACCCTCAAGGC
CGCTGCCGGGCCAGGACCAGGCGACCGGGGGTGGGGAAACCACTCCGGCCTCTTTGGGAAGGGCGGCCCT
GGCCCAGGAGAACCATTCAGGGACTATGTCGATAGATTTTACAAAACGCTGCGGTGA

ag2-ub

ATGCAAATCTTTGTAAAAACCCTGACCGGAAAGACCATCACCTTGGAAGTGGAACCTAGTGACACTATTG
AAAATGTTAAGGCTAAGATACAGGATAAAGAGGGGATCCCTCCTGACCAGCAACGGCTCATCTTTGCTGG
TAAACAGCTCGAGGACGGACGGACTCTGTCTGATTACAATATCCAGAAGGAAAGCACCCTACACCTGGTT
CTAAGACTGCGAGGTGTACGTACGTTTTCAAGGAACTCAACCCACGAGATGTACTACTCAACCGCCGTGA
CCGGAAATGGACCGGGGCCTGGCGGCAGAGGTGGCTGGTCATACTATGCCGCCAGCCGACCCGCCGTAGG
TCCTGGTCCAGGCTCTACTGCAGTCACTGGTAATATCGTTAATTCTGTTAATGTCCAATCACGTAAGCTA
TTAGCCCGGTTTGGTCCGGGACCAGGCGGATCAATCATGGACGTCATCACAAGGCGCGACCAAAGAGGTT
CCGGCGGCCAGGTGGTAACATATGCTCTCAATACCTTGACAAACATAAAAGTCCAACTGATCCGAATGAT
GGAGGGGGAAGGAGTAATAGGGCCAGGGCCGGGACATGAGCTTGTGATGAAAGATGGTCGCACCCTAGTA
GTGCCTAGTAGAGACCAAGATGAGCTTGGGCCTGGGCCTGGCTGGAGCGTTCGCGAAACTGCATCCCTAA
GCAAGGCCTACGGCCAAATGTGGCTTCTGTCCTACTTCCACCGGCGGGATCTGCGGACACTGGGACTCGC
AATAAACTCCGCCGTCCCTGTCGGTCCTGGCCCCGGTAGCCCTACCATGGGTCCAGCTACCCTGGGTCCA
GGACCCGGTCCATGGAATGCTAGGGAAGATGTAGTGAGAATGGCGATGACAGACACTACAGCATTTGGCC
AGCAGCGTGTATTCAAGGGTCCAGGGCCGGGTGTCCACCGTGATTGGTTCAACGACTTAGCCCTTCCTGG
CCCTGGACCTGGCGAGTTTGGAGTCGCCAAAGGGAGCAGAGCTATATGGTATATGTGGTTAGGAAGCCGA
TTTTTAGAGTTTGAAGCCCTCGGCTTCTTAAACGAGGGACCTGGCCCAGGTTCCAAAGGGGTTCTCCACA
CCATGTGGCACGTCACTAGGGGGGCAGCATTATCTGGCCCAGGCCCTGGAGGGGCTTCAGTTAGATCGAC
AACAGAATCAGGAAAGGTGATACCTGAGTGGTCTTCAAGGGCTAGTACAATGCCAGGACCTGGTCCTGGA
GCAAAGTTTGTGGCCGCCTGGACGCTAAAAGCCGCAGCCGGGCCGGGGCCTGGGGACAGAGGATGGGGCA
ACCATTCCGGTTTATTCGGAAAAGGAGGTCCAGGCCCTGGCGAGCCCTTCCGAGATTATGTGGACCGGTT
CTACAAAACCCTTCGATGA

ag4-ub

ATGCAAATCTTTGTAAAAACCCTGACCGGAAAGACCATCACCTTGGAAGTGGAACCTAGTGACACTATTG
AAAATGTTAAGGCTAAGATACAGGATAAAGAGGGGATCCCTCCTGACCAGCAACGGCTCATCTTTGCTGG
TAAACAGCTCGAGGACGGACGGACTCTGTCTGATTACAATATCCAGAAGGAAAGCACCCTACACCTGGTT
CTAAGACTGCGAGGTGTACGTACGTTCTCAAGAAATTCTACTCATGAGATGTATTATTCCACCGCCGTTA
CCGGCAATGGCCGGGGCGGATGGAGCTACTACGCCGCTTCCAGGCCAGCGGTAAGTACAGCAGTGACTGG
AAACATCGTCAACTCCGTGAACGTGCAGTCAAGGAAGCTCTTGGCAAGATTCGGAAGTATTATGGACGTG
ATTACGAGGAGAGATCAGCGTGGGTCAGGGGGTCAGGTTGTGACATATGCACTTAACACTTTGACCAACA
TAAAGGTCCAGCTCATCCGAATGATGGAAGGCGAGGGCGTAATCCACGAACTGGTTATGAAGGACGGACG

TACCCTTGTGGTGCCAAGCCGCGATCAGGACGAGTTGTGGTCAGTCCGCGAGACAGCCTCTTTATCCAAG
GCTTACGGCCAGATGTGGCTGCTGTCATATTTCCACCGCAGAGATCTGCGGACACTGGGGTTGGCCATCA
ATTCCGCGGTGCCAGTTTCCCCCACTATGGGACCGGCCACCCTACCATGGAACGCTCGCGAGGATGTGGT
GCGCATGGCCATGACGGACACAACCGCATTCGGACAGCAGCGCGTGTTCAAGGTGCATAGGGATTGGTTT
AATGACTTGGCTCTCCCTGAGTTCGGCGTCGCCAAAGGGTCAAGAGCTATCTGGTATATGTGGCTGGGTA
GCAGATTTCTTGAATTTGAGGCTTTGGGGTTTCTTAACGAATCCAAGGGCGTGCTGCATACTATGTGGCA
CGTCACCCGTGGAGCCGCATTGAGTGGGGCTTCGGTAAGATCCACAACTGAGTCCGGGAAGGTTATACCT
GAGTGGAGCAGCAGAGCCAGCACTATGCCAGGCCCTGGACCTGGGGCCAAATTCGTAGCTGCCTGGACCC
TCAAGGCCGCTGCCGGACCCGGCCCAGGCGACAGGGGATGGGGGAATCACTCCGGACTGTTCGGTAAAGG
AGGTCCCGGACCTGGAGAACCCTTCAGAGACTACGTGGATCGCTTCTACAAGACTCTGCGC
