# Supplementary material for: How Does Inter-Epitope Spacer Variation Within Artificial Immunogens Based on T-Cell Epitopes of Tick-Borne Encephalitis Virus Affect Immunogenicity?
Source: Vaccines (Basel). 2026 Sep 2;14(9):770. doi: 10.3390/vaccines14090770 (PMC13611838; doi:10.3390/vaccines14090770)

Figure S3. The whole electrophoregram of PCR analysis.

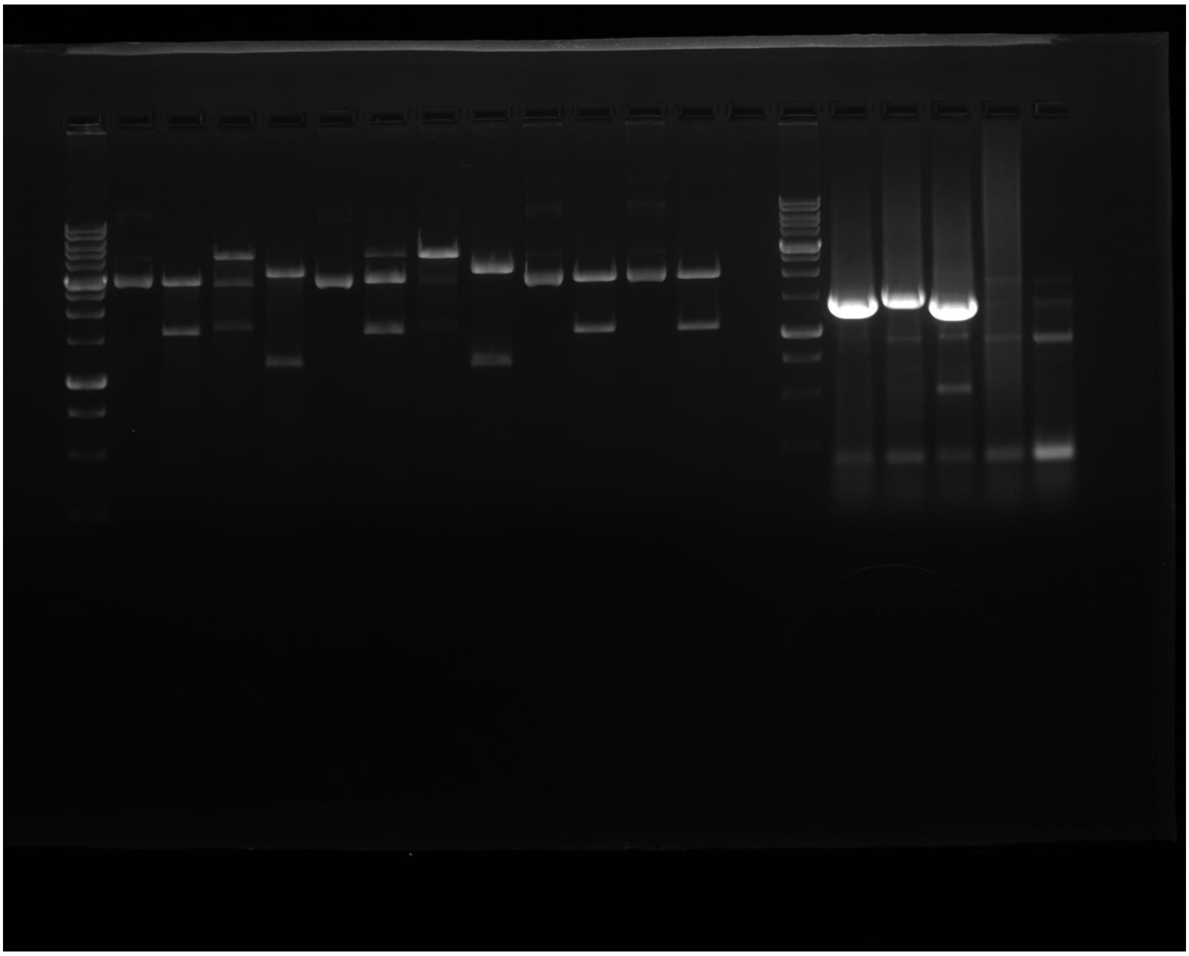

Supplement: Supplementary file 1 [file vaccines-14-00770-s001.zip › Supplementary Material Figure S3. The whole electrophoregram of PCR analysis.pdf]
