# Supplementary material for: How Does Inter-Epitope Spacer Variation Within Artificial Immunogens Based on T-Cell Epitopes of Tick-Borne Encephalitis Virus Affect Immunogenicity?
Source: Vaccines (Basel). 2026 Sep 2;14(9):770. doi: 10.3390/vaccines14090770 (PMC13611838; doi:10.3390/vaccines14090770)
Supplement: Supplementary file 1 [file vaccines-14-00770-s001.zip › Supplementary Material File S1. Densitometry of Western blot analysis.pdf]

iBright™ Image Analysis Report

8 August 2026

Western_blot

Date:	7 August 2026 07:01:54PM
Mode:	Tiff Import
Notes:	
iBA version:	5.6.0
Image size:	824px X 954px

AI600

Original file name:	Western_blot.tif
Image type:	Fluoro

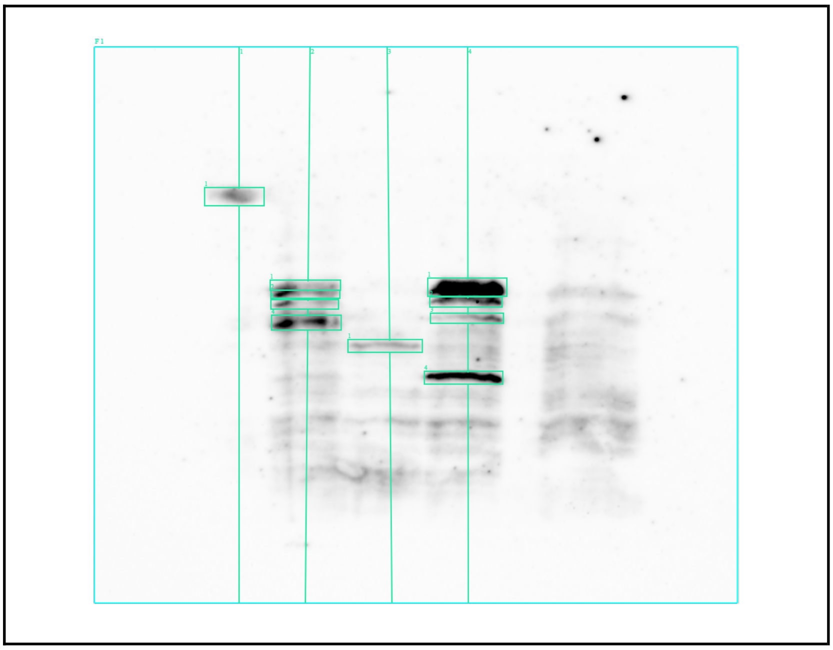

LANE AND BAND ANALYSIS DATA TABLE

Western_blot

Frame: 1
Channel: AI600
Sensitivity: 100

Lane 1

#	Vol. (Int.)	Local Bg. Corr. Vol.	Rolling Bg. Corr. Vol.	Area	Rf
1	32,897,285	24,645,089	26,430,464	2,492	0.269

Lane 2

#	Vol. (Int.)	Local Bg. Corr. Vol.	Rolling Bg. Corr. Vol.	Area	Rf
1	30,221,658	8,706,462	19,017,728	1,680	0.428
2	37,637,136	14,227,639	27,685,632	1,339	0.445
3	27,064,413	6,181,964	15,024,128	1,515	0.463
4	78,791,317	44,576,452	56,920,320	2,392	0.496

Lane 3

#	Vol. (Int.)	Local Bg. Corr. Vol.	Rolling Bg. Corr. Vol.	Area	Rf
1	27,347,884	14,146,725	17,780,480	2,220	0.537

Lane 4

#	Vol. (Int.)	Local Bg. Corr. Vol.	Rolling Bg. Corr. Vol.	Area	Rf
1	156,156,798	100,790,537	137,111,552	3,304	0.431
2	61,760,698	17,307,806	48,655,104	1,728	0.458
3	29,445,775	14,239,279	14,555,392	1,744	0.487
4	88,896,043	68,639,232	68,758,272	2,340	0.594
