# Supplementary material for: How Does Inter-Epitope Spacer Variation Within Artificial Immunogens Based on T-Cell Epitopes of Tick-Borne Encephalitis Virus Affect Immunogenicity?
Source: Vaccines (Basel). 2026 Sep 2;14(9):770. doi: 10.3390/vaccines14090770 (PMC13611838; doi:10.3390/vaccines14090770)
Supplement: Supplementary file 1 [file vaccines-14-00770-s001.zip › Supplementary Material File S2. Kruskal–Wallis test ELISpot with Dunn's multiple comparisons test.pdf]

File S2. Kruskal–Wallis test ELISpot with Dunn's multiple comparisons test

Number of families	1
Number of comparisons per family	15
Alpha	0,05

Dunn's multiple comparisons test	Mean rank diff,	Significant?	Summary	Adjusted P Value	
pVAX-AG1-ub vs. pVAX-AG2-ub	18	Yes	*	0,0263	A-B
pVAX-AG1-ub vs. pVAX-AG4-ub	0,6667	No	ns	>0,9999	A-C
pVAX-AG1-ub vs. pVAX1	18,42	Yes	*	0,0205	A-D
pVAX-AG1-ub vs. Commercial vaccine	19,33	Yes	*	0,0116	A-E
pVAX-AG1-ub vs. Naive	17,58	Yes	*	0,0335	A-F
pVAX-AG2-ub vs. pVAX-AG4-ub	-17,33	Yes	*	0,0387	B-C
pVAX-AG2-ub vs. pVAX1	0,4167	No	ns	>0,9999	B-D
pVAX-AG2-ub vs. Commercial vaccine	1,333	No	ns	>0,9999	B-E
pVAX-AG2-ub vs. Naive	-0,4167	No	ns	>0,9999	B-F
pVAX-AG4-ub vs. pVAX1	17,75	Yes	*	0,0304	C-D
pVAX-AG4-ub vs. Commercial vaccine	18,67	Yes	*	0,0176	C-E
pVAX-AG4-ub vs. Naive	16,92	Yes	*	0,0491	C-F
pVAX1 vs. Commercial vaccine	0,9167	No	ns	>0,9999	D-E
pVAX1 vs. Naive	-0,8333	No	ns	>0,9999	D-F
Commercial vaccine vs. Naive	-1,75	No	ns	>0,9999	E-F

Test details	Mean rank 1	Mean rank 2	Mean rank diff,	n1	n2	Z
pVAX-AG1-ub vs. pVAX-AG2-ub	30,83	12,83	18	6	6	3,129
pVAX-AG1-ub vs. pVAX-AG4-ub	30,83	30,17	0,6667	6	6	0,1159
pVAX-AG1-ub vs. pVAX1	30,83	12,42	18,42	6	6	3,202
pVAX-AG1-ub vs. Commercial vaccine	30,83	11,5	19,33	6	6	3,361
pVAX-AG1-ub vs. Naive	30,83	13,25	17,58	6	6	3,057
pVAX-AG2-ub vs. pVAX-AG4-ub	12,83	30,17	-17,33	6	6	3,013
pVAX-AG2-ub vs. pVAX1	12,83	12,42	0,4167	6	6	0,07244
pVAX-AG2-ub vs. Commercial vaccine	12,83	11,5	1,333	6	6	0,2318
pVAX-AG2-ub vs. Naive	12,83	13,25	-0,4167	6	6	0,07244
pVAX-AG4-ub vs. pVAX1	30,17	12,42	17,75	6	6	3,086
pVAX-AG4-ub vs. Commercial vaccine	30,17	11,5	18,67	6	6	3,245
pVAX-AG4-ub vs. Naive	30,17	13,25	16,92	6	6	2,941
pVAX1 vs. Commercial vaccine	12,42	11,5	0,9167	6	6	0,1594
pVAX1 vs. Naive	12,42	13,25	-0,8333	6	6	0,1449
Commercial vaccine vs. Naive	11,5	13,25	-1,75	6	6	0,3042
