# Supplementary material for: How Does Inter-Epitope Spacer Variation Within Artificial Immunogens Based on T-Cell Epitopes of Tick-Borne Encephalitis Virus Affect Immunogenicity?
Source: Vaccines (Basel). 2026 Sep 2;14(9):770. doi: 10.3390/vaccines14090770 (PMC13611838; doi:10.3390/vaccines14090770)
Supplement: Supplementary file 1 [file vaccines-14-00770-s001.zip › Supplementary Material File S3. Kruskal–Wallis test ELISA with Dunn's multiple comparisons test.pdf]

File S3. Kruskal–Wallis test ELISA with Dunn's multiple comparisons test

Number of families	1
Number of comparisons per family	5
Alpha	0,05

Dunn's multiple comparisons test	Mean rank diff,	Significant?	Summary	Adjusted P Value	E-?	
Commercial vaccine vs. pVAX-AG1-ub	17,93	Yes	**	0,009	A	pVAX-AG1-ub
Commercial vaccine vs. pVAX-AG2-ub	17,83	Yes	**	0,0095	B	pVAX-AG2-ub
Commercial vaccine vs. pVAX-AG4-ub	17,93	Yes	**	0,009	C	pVAX-AG4-ub
Commercial vaccine vs. pVAX1	18,5	Yes	**	0,006	D	pVAX1
Commercial vaccine vs. Naive	18,5	Yes	**	0,006	F	Naive

Test details	Mean rank 1	Mean rank 2	Mean rank diff,	n1	n2	Z
Commercial vaccine vs. pVAX-AG1-ub	33,5	15,67	17,93	6	6	3,121
Commercial vaccine vs. pVAX-AG2-ub	33,5	16,17	17,83	6	6	3,033
Commercial vaccine vs. pVAX-AG4-ub	33,5	15,67	17,93	6	6	3,121
Commercial vaccine vs. pVAX1	33,5	15	18,5	6	6	3,237
Commercial vaccine vs. Naive	33,5	15	18,5	6	6	3,237
